# Population Genomic Analyses Reveal Geographic Structure in *Rhizoctonia solani* AG1‐IA Isolates Associated With Agronomic Crops

**DOI:** 10.1111/mec.70359

**Published:** 2026-04-27

**Authors:** Juanita Gil, Kensy Rodriguez‐Herrera, Vanina Castroagudin, Felipe Dalla Lana, Camila P. Nicolli, Xin‐Gen Zhou, Sara Thomas‐Sharma, Terry Spurlock, Jim C. Correll, Pierre Gladieux, Alejandro Rojas

**Affiliations:** ^1^ Department of Entomology and Plant Pathology University of Arkansas Fayetteville Arkansas USA; ^2^ Department of Plant, Soil and Microbial Sciences Michigan State University East Lansing Michigan USA; ^3^ Department of Plant Pathology and Crop Physiology Louisiana State University AgCenter Baton Rouge Louisiana USA; ^4^ USDA‐ARS Mycology & Nematology Genetic Diversity & Biology Laboratory Beltsville Maryland USA; ^5^ University of Arkansas System Division of Agriculture Stuttgart Arkansas USA; ^6^ Texas A&M AgriLife Research Center Beaumont Texas USA; ^7^ University of Arkansas System Division of Agriculture Monticello Arkansas USA; ^8^ Plant Health Institute Montpellier, University of Montpellier INRAE, CIRAD, IRD, Institut Agro Montpellier France

## Abstract

Soilborne fungal pathogens threaten food security, but limited knowledge of their population biology, including genetic variability and population structure, hinders the development of effective strategies to prevent crop losses. *Rhizoctonia solani* AG‐1 is a significant soilborne pathogen worldwide, divided into subgroups based on host range and molecular diversity. *Rhizoctonia solani* AG1‐IA causes sheath blight in rice and aerial blight in soybean, two devastating diseases in these economically important crops. Rice and soybean are often grown in rotation, leading to the buildup of inoculum in the fields over cropping seasons. Monitoring the genetic variability and structure of *R. solani* AG1‐IA is critical to understand the population biology of the pathogen and aid in disease management practices, including the screening and development of new resistant cultivars. A total of 145 isolates of *R. solani* collected between 1993 and 2022 from different hosts and states in the USA were sequenced. The population genetic structure was inferred with clustering approaches based on approximately two million biallelic single nucleotide polymorphisms (SNPs). *Rhizoctonia solani* AG1‐IA showed relatively high genetic diversity and little departure from mutation‐drift equilibrium, suggesting that *R. solani* AG1‐IA is a well‐established, widespread pathogen with a stable demographic history in the USA. While populations had a clear geographic structure, they lacked host specialization, suggesting dispersal is mainly distance limited. Shared ancestry between populations and the discovery of clonal lineages, however, indicated recent connections between geographic areas. Our work provides valuable insights into the evolutionary history and population biology of *R. solani* AG1‐IA, offering a foundation for developing targeted management strategies and resistant crop varieties.

## 1 Introduction

Soilborne pathogenic fungi pose a significant threat to global food security (Delgado‐Baquerizo et al. 2020). However, our understanding of their population biology remains limited compared to the more widely studied airborne pathogens. Some key questions to be answered in the context of soilborne plant pathogenic fungi include characterizing their genetic diversity, which provides insights into the history and origin of pathogenic populations, and population structure, which informs our understanding of host specialization and the factors influencing dispersal (Gladieux et al. 2016; Hessenauer et al. 2021). For example, while airborne fungi have a long‐range distribution, with fungal communities being similar even at distances > 100 km, the dispersal of fungi in soil is limited and mostly attributed to anthropogenic factors in the case of agronomic plant pathogens (Termorshuizen 2016; Wagner et al. 2022). Moreover, abiotic parameters, latitude and pathogen persistence also play important roles in shaping the soil fungal community composition and genetic structure (Kivlin et al. 2014; Termorshuizen 2016). Certain physical soil properties and host density thresholds have been shown to be required for soilborne fungal epidemics (Otten and Gilligan 2006). Additionally, the cropping system is an important structuring factor in polyphagous soilborne fungal pathogen populations (Walker et al. 2015; Chavarro Mesa et al. 2015), increasing population sizes by allowing persistence on multiple hosts. Understanding the spatial distribution of genetic diversity and the population structure of soilborne fungal pathogens is important to study the evolutionary trade‐offs between host specialization and environmental plasticity in heterogeneous landscapes.

*Rhizoctonia solani* Kühn (Basidiomycota: Agaricomycetes) is a ubiquitous necrotrophic plant pathogen that affects over 200 different plant species, including those in the families *Poaceae* and *Fabaceae* (Ajayi‐Oyetunde and Bradley 2018). Rice and soybeans are staple foods of global importance, serving as key sources of nutrition for humans and animals, respectively. Worldwide, China and India are the leading rice producers, while the United States contributes less than 2% to the global rice production. Despite its lower production share, the USA is the fifth‐largest rice exporter (FAO 2018), cultivating approximately 1.25 million hectares of rice annually. The state of Arkansas accounts for 48% of the national rice production with nearly 600,000 ha planted, followed by California (17%), Louisiana (16%), Missouri (8%) and Texas (5%) (NASS 2024). Simultaneously, soybeans are the primary crop for animal feed, vegetable oil production and derived products for human consumption. The United States holds a significant role as the second‐largest soybean producer globally (USDA ERS—Soybeans and Oil Crops 2023), and Arkansas is the leading soybean‐producing state in the mid‐south (Ross 2022). In areas where both rice and soybeans can be grown, these two crops are often planted in rotation, creating a fluctuating selective environment that can increase the pressure of diseases caused by pathogens that have both plants as major hosts, like the soilborne Basidiomycete fungus *R. solani* .

*Rhizoctonia solani* is a species complex encompassing different lineages referred to as anastomosis groups (AGs). Thirteen AGs were identified (AG‐1 through AG‐13, and bridging group AG‐BI) based on the ability of compatible hyphae to fuse (Carling et al. 2002). Among these groups, AG‐1 is an important group that is further divided into intraspecific groups based on differences in host range and molecular diversity (Verwaaijen et al. 2017). In particular, *R. solani* AG1‐IA is considered a highly destructive pathogen causing sheath blight of rice, a disease that can lead to yield losses up to 50% (Zheng et al. 2013), aerial blight in soybean (40% to 50% yield loss) (Faske et al. 2014) and banded leaf blight in corn (up to 100% yield loss mainly in tropical areas) (Hooda et al. 2017). Moreover, *R. solani* develops sclerotia, remaining in a dormant stage in the soil for many years, which confers a fitness advantage that allows the fungus to germinate once conditions are favourable and a suitable host is present. Currently, there is limited resistance to sheath blight in rice (Shi et al. 2021), and no commercial soybean cultivars are known to be resistant to aerial blight (Harville et al. 1996; Rodriguez‐Herrera et al. 2023, 2024), making the incidence of the pathogen in these cropping systems a major problem for crop production.

Anthropogenic pressures, including the frequent and widespread use of fungicides and monoculture rotations, impose strong selective pressures that shape the high genetic variability of *R. solani* AG1‐IA and the adaptive landscape of its genome (Chen et al. 2022; Senapati et al. 2022). While previous studies on population structure and genetic variability among isolates of *R. solani* AG1‐IA established the presence of high levels of genetic diversity and suggested host specialization using neutral SSR markers (e.g., Ciampi et al. 2008; de Assis et al. 2008; Rosewich et al. 1999; Taheri et al. 2007; Wang et al. 2013), they lacked the genomic resolution to distinguish between neutral population structure and adaptive differentiation. Furthermore, most recent genomic efforts have focused exclusively on single‐hosts: rice‐infecting populations in Asia (Cumagun et al. 2019; Chen et al. 2022; Francis et al. 2023; Kumar et al. 2021; Tian et al. 2022) or soybean populations in the USA (Rodriguez‐Herrera et al. 2024). In this study, we used a population genomics approach to test whether host‐driven selection or geographic isolation is the primary driver of divergence in *R. solani* AG1‐IA. By analysing a dataset of approximately 2 million high‐quality single nucleotide polymorphisms (SNPs), we assess the recombination landscape and identify genomic regions under positive and balancing selection, providing insights into the mechanisms that maintain niche breath in a ubiquitous soilborne generalist. Furthermore, characterizing the genetic variation and host‐pathogen dynamics in *R. solani* AG1‐IA provides a critical framework for predicting how this pathogen responds to anthropogenic activities, such as crop rotation and regional seed movement. Integrating these genomic insights into breeding programs and disease management is essential for developing durable resistance and sustainable, ecology‐based agricultural solutions that safeguard global food staples in a changing environment.

## 2 Materials and Methods

### 2.1 Collection of *Rhizoctonia solani* AG1‐IA Isolates

Isolates of *R. solani* belonging to anastomosis group AG1‐IA were collected from different plant hosts and geographical regions at the dikaryotic stage (i.e., cells contained two genetically distinct nuclei, one from each parent). Isolates were observed under the microscope and major allele frequencies calculated to determine the dikaryotic stage. Isolates collected between 2020 and 2022 were obtained from symptomatic rice and soybean leaves showing symptoms of sheath and aerial blight diseases, respectively. The plant tissue was collected in Ziploc bags across 17 fields in Arkansas, brought to the laboratory and stored at 4°C. Fungal isolation was achieved by cutting symptomatic tissue into small pieces, surface‐sterilizing the tissue with 70% ethanol and sterile water by immersing the tissue pieces in each solution for 1 min and plating them on potato dextrose agar medium (PDA) (Hardy Diagnostics, Santa Maria, CA) amended with antibiotics (0.075 mg/mL streptomycin sulfate). Isolates from 2009 and earlier years were obtained from laboratory collections at the Department of Entomology and Plant Pathology, University of Arkansas. These were collected from infected host tissue, field soil or sclerotia from rice debris and isolated as described in Castroagudin (2012). More recent isolates from Texas were obtained from research trials as described in Uppala and Zhou (2018) and Zhou et al. (2021) or nearby rice fields, and Louisiana isolates were obtained from commercial fields with natural infections (Rodriguez‐Herrera et al. 2024). Anastomosis group classification was done by amplifying and sequencing the ITS region using primers ITS1F and ITS4 and running a phylogenetic analysis. In total, 145 *R. solani* isolates were analysed and obtained from the following hosts: rice (*n* = 76), soybean (*n* = 65), corn (*n* = 1), sorghum (*n* = 1), Bermuda grass (*n* = 1) and common bean (*n* = 1). Approximately the same number of isolates were collected from Arkansas (*n* = 66) and Louisiana (*n* = 62), while 16 isolates were collected in Texas, and one originated from Cuba. All isolates and their corresponding information are listed in Table S1, and the geographical distribution of the samples is shown in Figure S1.

### 2.2 DNA Extraction and Sequencing

All isolates were grown on potato dextrose agar (PDA) (Hardy Diagnostics, Santa Maria, CA) media and transferred to ¼ strength potato dextrose broth (PDB) for mycelial mat production. Flasks with PDB were inoculated with four mycelial plugs and incubated as static cultures at room temperature. Mycelia were harvested after 1 week of growing in liquid culture by filtering the media using Büchner funnels (VWR International LLC, Radnor, PA) and vacuum suction. Mycelia were washed twice with sterile water to remove excess media. The dry mycelia collected on sterile filter paper were then transferred into sterile 2 mL Eppendorf tubes and stored at −20°C until further processing. Mycelia were lyophilized for 24 h and ground to a fine powder in a 1600 Spex MiniG tissue homogenizer at 1500 rpm for 3 min using 3.2 mm stainless steel beads. Genomic DNA (gDNA) was extracted using the E.Z.N.A. HP Fungal DNA Kit (Omega Bio‐Tek, Norcross, GA) following the manufacturer's instructions. The quality of the gDNA was assessed using agarose gel‐based electrophoresis and UV–Vis measurements on a ND‐1000 Nanodrop spectrophotometer (ThermoFisher Scientific, Waltham, MA), while the total DNA concentration was quantified by Qubit (ThermoFisher Scientific, Waltham, MA) using the 1× dsDNA HS assay. The obtained gDNA was sequenced by Admera Health, South Plainfield, NJ, USA via the Illumina technology on a NovaSeq 6000 S4 machine producing 2 × 150 bp reads and 10 million PE reads per sample.

### 2.3 Reference Genome and Read Alignment

All reads of all samples were analysed using FastQC v.0.11.9 (Andrews 2019) and MultiQC (Ewels et al. 2016) for sequencing quality check. Only samples with detectable adapters were modified; that is, Illumina adapters were removed using Cutadapt v.4.0 (Martin 2011). To avoid keeping empty reads (default behaviour of Cutadapt), a minimum read length filter of 40 bp was used. The assembly of *R. solani* AG1‐IA isolate HG81 isolated from rice in Huanggang, Hubei, China (Yang et al. 2022) was used as a reference genome and downloaded from the NCBI database (BioSample SAMN28614015). For all samples with 0% adapter content, raw reads were mapped to the reference genome using bowtie2 v.2.4.5 (Langmead and Salzberg 2012); otherwise, trimmed reads after adapter removal were mapped. Most parameters were used with their default values, except for the maximum number of alignments per read, which was set to three, and the maximum fragment length, which was set to 1000. Alignments in the SAM files were then sorted by reference coordinates and converted to BAM files using Picard (https://broadinstitute.github.io/picard/).

### 2.4 Variant Detection, Genotyping and Filtering

Before variant calling, the major allele frequencies were calculated for each isolate in the population using previously obtained VCF files to estimate their ploidy (Figure S2). Variant calling was then performed using mpileup in bcftools (Li 2011), with the multiallelic calling model and setting the ploidy to two. Distributions of QUAL values, mapping quality and read depth were calculated and used to filter out variants according to the following values: minimum quality > 5, minimum mapping quality > 10 and minimum read depth > 10. Small indels were also removed. Sites with more than two alleles or with missing data for more than 10% of the isolates were masked (i.e., considered as missing data). The population VCF file was annotated using the VCFAnnotate module from NGSEP v.4.3.1 (Gonzalez‐Garcia et al. 2023) to identify the type of variants called using the annotation file of the reference genome. The final VCF included both monomorphic and polymorphic sites to ensure an accurate estimation of genomic variability (Korunes and Samuk 2021).

### 2.5 Clone Correction

Clonality in our set of *R. solani* AG1‐IA isolates was assessed with the R package Poppr v.2.9.4 (Kamvar et al. 2014). First, the final population VCF file containing only biallelic SNPs was uploaded into R and then converted to genind and genlight objects. Metadata were also uploaded and used to assign strata. Then, a PCA analysis was conducted and finally, clonality was assessed as follows: (1) the number of multilocus genotypes (MLGs) was calculated. Initially, an MLG was defined as a unique combination of SNPs in the set of filtered variants. (2) To account for missing data and genotyping errors, MLGs were determined based on genetic distances and collapsed into larger groups called multilocus lineages (MLLs) using the farthest neighbour algorithm. (3) Genetic distances were calculated as the dissimilarity (i.e., Hamming) distance between SNPs. (4) The threshold to determine the minimum genetic distance at which two individuals would be considered to come from different clonal lineages was estimated by the function cutoff predictor in Poppr. (5) Clone correction of the sample set was performed by randomly sampling a single representative from each clonal lineage.

### 2.6 Assessment of Population Structure

The final population VCF file was converted to a FASTA file using a custom python script (vcf2fastadiploid.py) that maps each genotype (or pair of alleles) to their official IUPAC codes. This file was uploaded to SplitsTree v.4 (Huson 1998) to generate a phylogenetic network and examine the relationships between the isolates. Individual ancestry coefficients in *K* ancestral populations were inferred using the program sNMF (Frichot et al. 2014). Parameter *K* was set from 1 to 10, and 10 repetitions for each *K* value were calculated. The entropy parameter was set to true to calculate the cross‐entropy criterion. Based on this criterion, the final *K* value (*K* = 2) was selected by calculating the lowest cross‐entropy value in all runs. Ancestry coefficients were visualized using the R package pophelper (Francis 2017).

### 2.7 Genomic Variation in Subpopulations

Summary statistics of genomic variation and population differentiation including nucleotide diversity within (*π*) and between populations (*d*_*xy*_), *F*_ST_ and Tajima's *D* were calculated using the tools pixy v.1.2.8 (Korunes and Samuk 2021) and scikit‐allel v.1.3.8 (Miles et al. 2024) on 10 Kbp genomic windows using the final population VCF file that included both monomorphic and polymorphic sites. An analysis of molecular variance (AMOVA) was conducted to further quantify population differentiation using the AMOVA function in the R package Poppr. Only the two main subpopulations (Arkansas and Louisiana) and hosts (rice and soybean) were included to ensure stable variance components. Population hierarchy was set to host within location, clone correction was set to TRUE to remove potential bias caused by cloned genotypes, and the threshold to differentiate MLGs was set to 0.03. Statistical support was obtained using the randtest function in R with 999 repetitions.

### 2.8 Test of Balancing Selection Based on Synonymous and Nonsynonymous Diversity

Signatures of multiallelic balancing selection were investigated based on levels of nonsynonymous to synonymous nucleotide diversity. First, pseudosequences for coding regions of all genes were generated using the VCF file and the annotation file (i.e., GFF) of the reference sequence. Then, summary statistics of synonymous and nonsynonymous variation were computed using EggLib v.3.0 (Siol et al. 2022), allowing for a maximum proportion of missing data of 0.5, a minimum gene length of 400 bp, and a minimum sample size of 10.

### 2.9 Linkage Disequilibrium, Recombination and Genome Scan for Selective Sweeps

Linkage disequilibrium (LD) (*r* ^2^) was measured in each subpopulation using PopLDdecay v.3.42 (Zhang et al. 2019) with default parameters corresponding to a maximum distance between two SNPs of 300 Kb, a minor allele frequency of 0.005, a maximum ratio of heterozygous allele of 0.88 and a maximum ratio of missing allele of 0.25. LD decay was plotted using the script Plot_MultiPop.pl, which is included in PopLDdecay.

Signatures of recent directional selection (i.e., selective sweeps) along the genomes were estimated using selscan v.2.0.0 (Szpiech 2024), implementing the Cross‐population Extended Haplotype Homozygosity (XPEHH) algorithm (Sabeti et al. 2007) using phased genotypes. Phasing of the genotypes in each subpopulation was performed with beagle v.5.4 (Browning et al. 2021) for each chromosome in the reference genome. For the cross‐population analysis in selscan, the Arkansas subpopulation was used as the reference and the Louisiana subpopulation as the target. The cutoff to stop the EHH decay was set to 0.2 and physical distances were used for the EHH computation. Genome‐wide normalization of the XPEHH scores was carried out with norm v.1.3.0 with the flag ‐‐xpehh. The analysis was also performed by windows using the flags ‐‐bp‐win and ‐‐winsize 10000. After normalization, consecutive windows in the top 0.5% were merged and genes within these regions were identified.

### 2.10 Over‐Representation Analysis of Genes in Regions Under Positive Selection

To study gene classes overrepresented in three different sets of genes under positive selection, namely genes with signatures of balancing selection based on synonymous and nonsynonymous diversity, genes with signatures of divergent selection based on *F*_ST_ values, and genes with signatures of recent directional selection based on haplotype homozygosity, an over‐representation analysis was performed using the R package ClusterProfiler (Yu et al. 2012). Gene ontology (GO) terms were used for the analysis. The adjusted *p*‐value cutoff was set to 0.1 and false discovery rate (fdr) was used as the correction method.

## 3 Results

### 3.1 Genomic Variation

We obtained an average of 15 million paired‐end reads per isolate in the analysed population. Assessment of raw sequences indicated a high quality, showing mean quality Phred scores > 30. However, Illumina adapters were detected in 20 out of the 145 sequenced isolates failing the adapter content check and were removed from the sequences before mapping to the reference genome. By removing Illumina sequencing adapters and filtering out reads shorter than 40 bp in length, between 300 and 40,000 reads were lost per sample, representing only up to 0.3% of the original data. This means 99.7% of the reads remained untouched after the trimming step, indicating minimal adapter contamination. Although the minimum read length was set low, the distribution of read lengths after trimming showed that reads were > 140 bp long based on FastQC and MultiQC analyses. The genome size of the selected reference isolate was 41.4 Mb (Yang et al. 2022) yielding average genome mapping coverage estimates that ranged from 33× to 154× across isolates. Overall alignment rates ranged from 82.9% to 91.5%, which are consistent with mapping rates reported in previous high‐quality studies for isolates of the AG1‐IA subgroup of *R. solani* (Francis et al. 2023). Isolates with alignment rates below 80% (*n* = 15) were excluded from further analyses. Removing these isolates from the population did not affect its composition because those lower alignment rates occurred in samples across all subpopulations. The highest mapping percentages did not necessarily correspond with isolates obtained from the same host (rice) as the reference genome. We observed the highest overall alignment rates (90.3%–91.5%) in three isolates collected from soybeans in Arkansas.

In total, 2.76 million biallelic variants were called in the *R. solani* AG1‐IA population. After filtering by quality scores > 5, mapping quality scores > 10, read depths > 10, minimum and maximum alleles per site of 2, a maximum of 10% of missing data per site and after removing clonal isolates, a total of 1,966,492 biallelic SNPs were retained for posterior analyses.

### 3.2 Population Subdivision and Clonality

**FIGURE 1:**
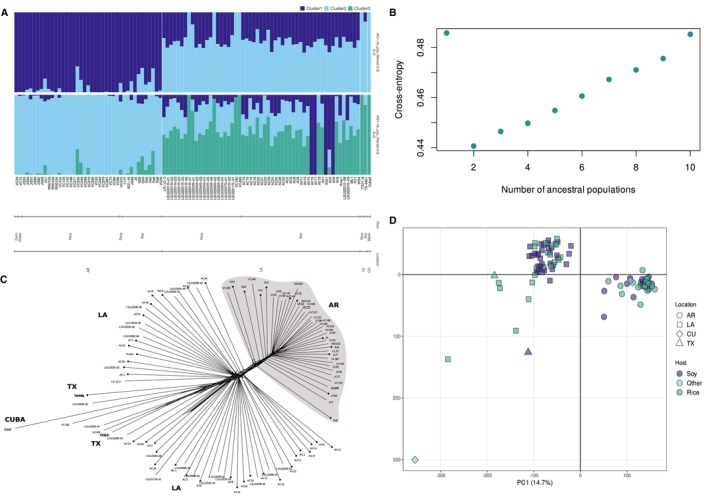
Population structure analysis inferred based on single nucleotide polymorphisms identified in 99 isolates of *Rhizoctonia solani* AG1‐IA from Arkansas (AR), Louisiana (LA), Texas (TX) and Cuba (CU) and six different hosts (rice, soybean, corn, grass, sorghum and common bean). A single representative of each clonal lineage was included in the analysis (i.e., the dataset was clone‐corrected). (A) Ancestry proportions in *K* clusters. Each bar represents a multilocus lineage and is divided into *K* segments representing membership in each cluster. (B) Cross‐entropy as a function of the number of (*K*) of 1 to 10 ancestral populations modelled. (C) Phylogenetic network with reticulations indicating phylogenetic conflicts caused by recombination, recurrent mutations or incomplete lineage sorting. Branches with dotted nodes represent soybean isolates. (D) Principal component (PC) analysis showing the first two PCs contributing to the population differentiation.

Population structure and ancestry proportions in *R. solani* AG1‐IA were estimated based on the model‐free clustering algorithm sNMF. The largest drop in cross‐entropy was observed between *K* = 1 and *K* = 2, and models with *K* > 2 did not identify well‐delimited clusters (Figure 1A,B and Figure S3). We, therefore, considered *K* = 2 as the most biologically relevant model of population subdivision. Location and host were two of the factors explaining the observed genetic differentiation, whereas the sampling year had little correlation with patterns of population subdivision (Figure S4).

The phylogenetic network computed with clone‐corrected data showed two main subpopulations, with one comprised of all the isolates from Arkansas (*n* = 44), and the other consisting of isolates from Louisiana, Texas and Cuba (*n* = 55) (Figure 1C). The host was less of a structuring factor than geography, although rice isolates from Texas and a small group of rice isolates from Louisiana showed very close genetic relationships (Figure S5A).

A principal component analysis (PCA) showed the same results as those observed with the phylogenetic network and clustering analysis. Principal component 1 explained 14.7% of the variation and principal component 2 explained 4.7% (Figure 1D). The population was primarily divided by geographical origin, with most isolates from Arkansas clustering together, and the remaining isolates forming a separate cluster. Isolates from Arkansas were also clustered very closely together, while the dispersion of the data among the other isolates was greater. The analysis of molecular variance on the clone‐corrected dataset further confirmed the strong geographic component of population subdivision, as the proportion of variation distributed between locations was relatively high and statistically significant (12.5% of variance, *p* = 0.001) (Table S2 and Figure S6).

**FIGURE 2:**
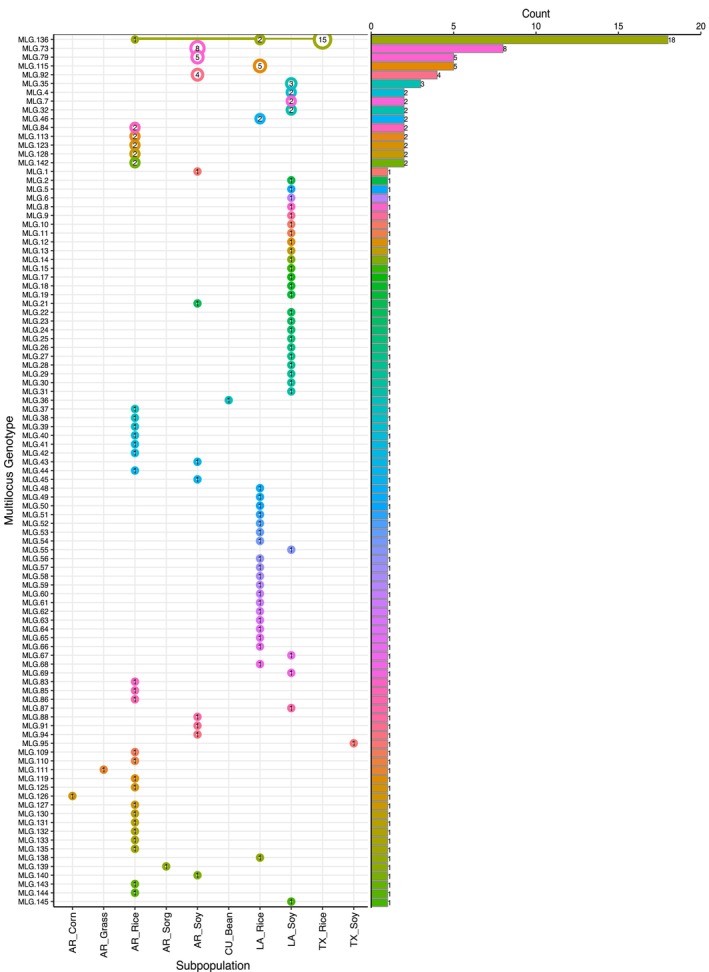
Distribution of multilocus genotypes (MLGs) of *Rhizoctonia solani* AG1‐IA among populations. MLGs are defined based on location and host. Unique MLGs (i.e., MLLs) detected in the population are shown in decreasing abundance highlighted by the bar plot (right) and the size of the circles on the dot plot (left). Each numbered circle represents the number of observations of each MLG and the lines connect genotypes found in different subpopulations.

To characterize the extent of clonality in *R. solani* AG1‐IA, multilocus genotypes (MLGs) and multilocus lineages (MLLs) were identified. Given the high resolution provided by ~2 million SNPs, every isolate was assigned to a unique MLG. However, the farthest neighbour algorithm using SNPs bitwise comparison of each genomic site classified the 145 isolates into 99 MLLs, resulting in 46 isolates being identified as clones in the population. A bimodal distribution of the genetic distances is expected if clones are present in the population, with a peak at lower values (< 0.03) corresponding with clonal isolates, while distances above the cutoff value represent unique MLGs. Each MLL encompassed one to 18 isolates or MLGs from the same location. Only the largest group (MLG.136) included genotypes from remote locations (Figure 2), grouping rice‐infecting isolates from Arkansas (*n* = 1), Louisiana (*n* = 2) and Texas (*n* = 15) in the same clonal lineage. This is consistent with long‐distance dispersal, potentially mediated by contemporary agricultural practices. All clonal isolates corresponded to the same host and, except for MLG.136, to the same location as well (Figure S5B).

### 3.3 Genomic Distribution of Polymorphism and Divergence in Arkansas and Louisiana Subpopulations

**FIGURE 3:**
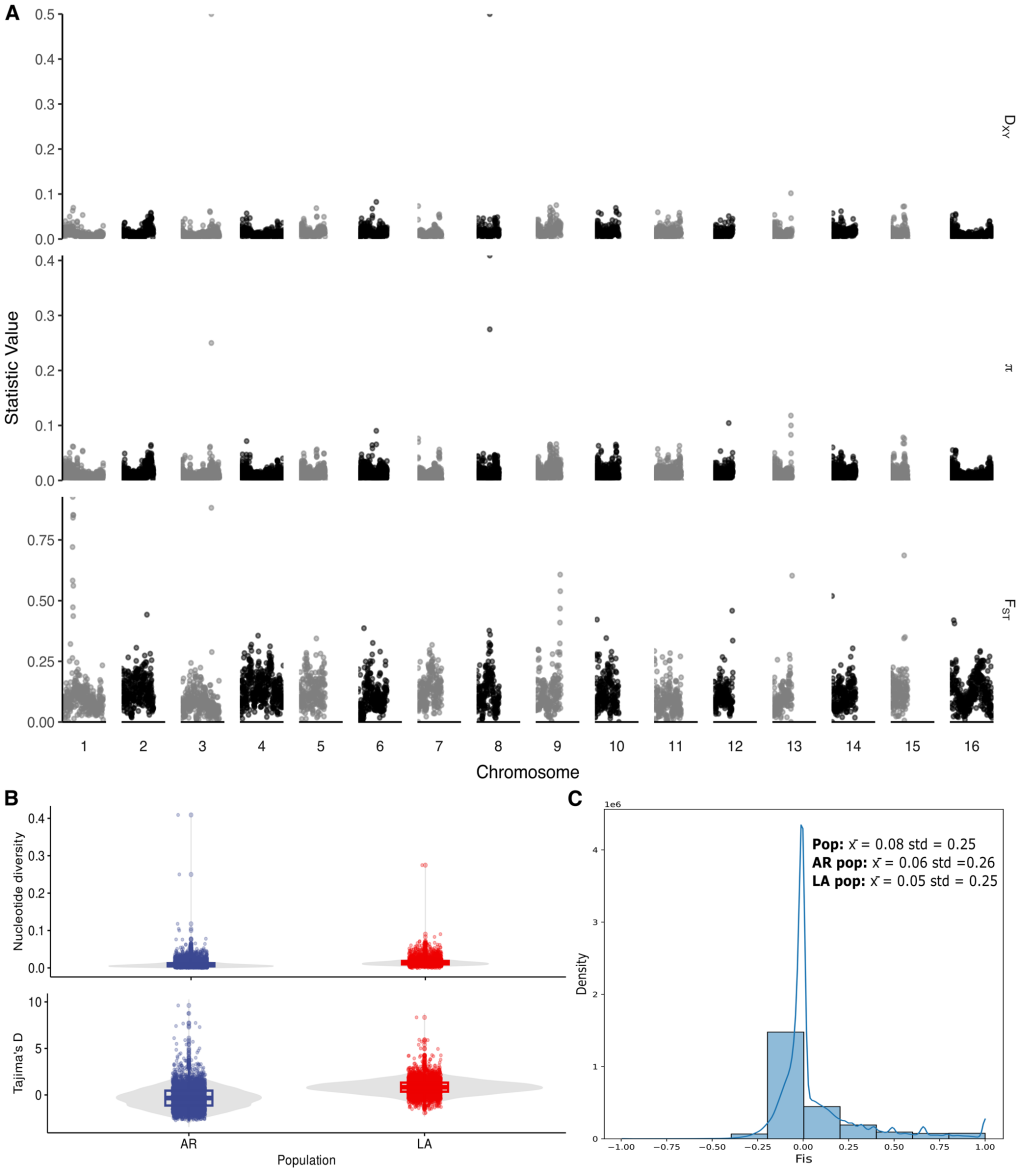
Distributions of nucleotide diversity and divergence within and between subpopulations of *Rhizoctonia solani* AG1‐IA. (A) Nucleotide diversity (*π*), absolute divergence (*d*_*xy*_) and relative divergence (*F*_ST_) along chromosomes. (B) Average nucleotide diversity (*y* axis cropped at 0.15) and Tajima's *D* by subpopulation, namely AR (Arkansas) and LA (Louisiana). (C) Distribution of the inbreeding coefficient in the entire population (pop) with mean values and standard deviations also reported for the Arkansas (AR) and Louisiana (LA) subpopulations.

Diversity and divergence statistics were calculated genome‐wide in 10 Kbp windows and are shown across all 16 chromosomes in the reference genome (Figure 3A). Regions of high absolute divergence between and diversity within subpopulations were observed on chromosomes 3 and 8, as indicated by peaks in *d*_*xy*_ and *π* values, respectively, but moderate values of *F*_ST_ (Figure 3A). Additionally, several chromosomes exhibited regions of high relative divergence between subpopulations, as indicated by peaks of *F*_ST_ (lower panel of Figure 3A). The top 5% of *F*_ST_ values across all windows of all chromosomes encompassed a total of 86,628 SNPs and 419 genes. This set of genes was enriched in three GO categories, representing 45: protein targeting (*n* = 18 genes), establishment of protein localization to organelle (*n* = 19), and protein targeting to vacuole (*n* = 8) (Figure S7). One of the genes (FUN_009043) encodes a catalytic component of the signal peptidase complex, which catalyses the cleavage of the N‐terminal signal sequences of proteins targeted to the endoplasmic reticulum.

The average nucleotide diversity was comparable between the Arkansas and Louisiana subpopulations (*π* = 0.012) (upper panel Figure 3B). However, despite similar levels of standing variation, the average values of Tajima's *D* differed between subpopulations, suggesting distinct demographic histories. While the mean values of *D* were not significantly different from zero in the Arkansas subpopulation, suggesting a stable demographic history, the Louisiana subpopulation showed a positive Tajima's *D*, indicating an excess of intermediate frequency alleles (lower panel Figure 3B). This excess of intermediate frequency alleles was consistent with the greater variance of the Louisiana subpopulation along the second component of the PCA (Figure 1D), suggesting population subdivision. The distribution of the inbreeding coefficient (*F*_IS_) peaked close to *F*_IS_ = 0, with a small tail towards *F*_IS_ = 1 resulting from sites with excess homozygosity (Figure 3C). The average *F*_IS_ value for the full dataset was 0.08. The average *F*_IS_ values for the Arkansas and Louisiana subpopulations, after clone correction, were 0.06 and 0.05, respectively. These *F*_IS_ estimates are consistent with high levels of outcrossing in *R. solani* AG1‐IA subpopulations.

### 3.4 Genes Under Multiallelic Balancing Selection

Signatures of multiallelic balancing selection in the Arkansas and Louisiana subpopulations were identified based on the ratio of nucleotide diversity at synonymous and non‐synonymous sites (*π*_N_/*π*_S_ > 1). Using this diversity ratio as a preliminary screen, we found 567 candidate genes with signatures of balancing selection. Of these, only 55 genes were common between the two subpopulations. Functional annotations were found for 235 genes, and the overrepresentation analysis did not reveal any Gene Ontology term that was more prevalent than expected in this dataset.

To investigate the efficiency of purifying selection in the subpopulations, the average of the *π*_N_/*π*_S_ ratios of all genes, excluding those with values higher than one, was calculated. The average for the Arkansas subpopulation was 0.319 and for Louisiana 0.239, suggesting that negative selection is efficiently acting to remove deleterious variants in these subpopulations, consistent with the relatively high effective population size reflected in *π* estimates.

### 3.5 Linkage Disequilibrium and Selective Sweeps

Blocks of linkage disequilibrium in *R. solani* AG1‐IA were very short. LD estimates as a function of physical distance showed a rapid and sharp decline, reaching half of the maximum *r* ^2^ value at the inter‐locus distance of 900–1000 bp (Figure S8). Signatures of selective sweeps across the genome and between the two subpopulations were identified based on haplotype homozygosity. After genome‐wide normalization, only windows in the top 0.5% were selected as candidate regions for selective sweeps on all chromosomes. A total of 271 regions were identified. Of those, 139 were defined for the Arkansas subpopulation and 132 for the Louisiana subpopulation. These regions comprised a total of 1.6 Mbp. Within these regions, 566 genes were identified, comprising 377 from the Arkansas subpopulation and 189 from the Louisiana subpopulation. Functional over‐representation analysis showed that only one GO term, namely MUB1‐RAD6‐UBR2 ubiquitin ligase complex, was represented more than expected in the dataset containing genes in selective sweeps regions in the Louisiana subpopulation. Three genes were associated with this category and all of them had a zinc finger MYND domain.

## 4 Discussion

*Rhizoctonia solani* AG1‐IA is the most destructive fungal pathogen on rice crops globally, causing the devastating disease of sheath blight (Savary et al. 2019). On soybeans and other crops, the pathogen can also cause important economic losses, and it is particularly severe in fields where rice and soybeans are planted in rotation. Rice and soybean crops lack cultivars with acceptable levels of resistance. Despite the availability of labelled fungicide methods for disease control, there is a risk of developing resistance to specific chemistries, making disease management challenging (Harville et al. 1996; Li et al. 2021; Rodriguez‐Herrera et al. 2023, 2024). Sensitivity to fungicides in *Rhizoctonia solani* AG1‐IA has been tested under laboratory conditions (Chen et al. 2022), and the effectiveness of fungicides to control sheath blight in rice has been tested under controlled field conditions (Uppala and Zhou 2018; Zhou et al. 2021). Here, we report on the population genomics of *R. solani* AG1‐IA, as inferred from the genomes of isolates obtained over three decades from six different hosts, primarily rice and soybeans, and four locations, including three within the USA.

The *R. solani* AG1‐IA populations in the USA exhibit relatively high levels of standing genetic variation. This has been previously demonstrated by other authors (e.g., Rosewich et al. 1999; Rodriguez‐Herrera et al. 2024), who found high diversity in populations in Texas and Louisiana associated with rice and soybeans, respectively. The fact that the total read alignment was approximately 90% may reflect some genomic divergence between the isolates studied here and the reference genome, and suggest that the Chinese population from which the reference genome was derived may represent a different lineage of *R. solani* AG1‐IA. While a divergent reference can lead to an underestimation of diversity by failing to map highly polymorphic reads (reference bias), it may also locally overestimate diversity if reads are misaligned to paralogous regions. However, we also observed similar mapping rates when using the B2 strain from the USA as the reference (Figure S9), indicating that the observed genomic differences among intraspecific *R. solani* isolates reflect intrinsic natural diversity rather than biases introduced by the choice of reference genome. The level of diversity in *R. solani* AG1‐IA in the USA is comparable to that reported in the textbook example of an invasive pathogen with high diversity, the wheat pathogen *Zymoseptoria tritici*, and far higher than many older invasive pathogens that are widely distributed, such as the ascomycetes *Blumeria graminis*, *Pyrenophora teres* or *Pyricularia oryzae*, or the basidiomycete *Puccinia striiformis tritici*. However, allele frequencies, as summarized by Tajima's *D*, do not reveal a signature of demographic expansion, unlike the previously mentioned pathogens. This supports the hypothesis of a local origin for the pathogen, rather than it being invasive. As with *Z. tritici*, sexual reproduction likely also contributes to maintaining diversity over time.

Population subdivision in *R. solani* AG1‐IA from the USA appears to be primarily explained by geography, rather than the host of origin of the samples. This supports that *R. solani* AG1‐IA is a host generalist and that our studied population lacks host specialization, but contrasts with the results by de Assis et al. (2008), who found that the fungal populations infecting rice and soybean in Louisiana are genetically distinct using 10 SSR markers. These contrasting results suggest that our whole‐genome data is providing a more nuanced view of the underlying genomic similarity between rice‐ and soybean‐infecting populations, although it is possible that some specific genes are specialized. Moreover, our genomic resolution might also be revealing a different trend in recent years. The importance of shared ancestry in clustering analyses indicates that differentiation between the two geographic subpopulations (Arkansas vs. Louisiana) is recent and/or that gene flow is significant between the two regions. Interestingly, our Texas isolates (*n* = 15) consisted of a single clonal lineage (MLG.136), contrasting with previous reports of high genetic diversity and random mating in Texas populations (Rosewich et al. 1999). This discrepancy stems from the origin of our Texas subset, which was collected from research plots rather than a broad survey of natural field populations. The observed clonality is therefore a result of the sampling context rather than a lack of state‐wide diversity. Nevertheless, the observation of the same clonal lineage in Arkansas, Louisiana and Texas supports the hypothesis of recent long‐distance migrations, likely via the movement of infested soil, seed or research materials, persistent sclerotia moving via irrigation or bodies of water, basidiospores dispersed by wind and even exchange of isolates between researchers. Long‐distance dispersal attributed to human‐mediated movement of infected planting materials, and potentially migratory birds, was discussed by Cumagun et al. (2019). These authors found unidirectional historical gene flow from Japan to China and from China to the Philippines following the movement of contaminated seeds and the widespread adoption of the Japanese japonica rice cultivar ‘Nongken 58’ in China during the 1950s and 1980s. Quantifying migration patterns and the direction of gene flow within the US populations will be essential to resolving the ongoing debate in fungal ecology regarding the degree of host specialization in *R. solani* AG1‐IA. Future genomic studies should employ uniform transect sampling, matched host‐specific sampling in shared environments, and broader multi‐continental scales to capture the full nuance of the pathogen's evolutionary trajectory. Furthermore, the observed differentiation between lineages could also have a climatic component. For instance, the higher clonal fraction in the Arkansas subpopulation might indicate that the warmer climate and milder winters in Louisiana, Texas and Cuba promote sexual reproduction of the pathogen, while colder temperatures in Arkansas might not be as conducive to this, leading to predominantly asexual reproduction in that region.

Louisiana and Arkansas subpopulations showed extensive shared ancestry, limited differentiation and shared clonal lineages, indicating that the two subpopulations have been connected by recent gene flow. In this context of low differentiation, the analysis of divergence in genomic windows allowed us to identify regions that resist migration between subpopulations (i.e., *F*_ST_‐outliers regions), possibly under the influence of divergent selection. Our genome‐wide scans for selection reveal evolutionary patterns that both complement and contrast with recent findings in Indian populations (Francis et al. 2023). While we identified significant selective sweeps encompassing 566 genes (top 0.5% of windows), Francis et al. primarily utilized Tajima's *D* to identify regions of purifying and diversifying selection across 42 Indian isolates. *F*_ST_‐outliers regions showed an overrepresentation of genes involved in protein sorting along secretory pathways and the process of targeting them to particular regions of the cell, particularly the vacuole. One of the 45 overrepresented genes in these regions was found to be a component of the signal peptidase complex, which has been identified as an important factor not only for normal vegetative growth but also for virulence and secretion of pathogenicity‐related extracellular enzymes in other fungi (Yang et al. 2023). This gene catalyses the signal peptide cleavage, which occurs during translocation of the mature protein through the translocon pore into the endoplasmic reticulum. Similarly, Francis et al. (2023) highlighted the importance of a glucosamine phosphate N‐acetyltransferase (GNAT) under purifying selection and a lytic polysaccharide monooxygenase (LPMO_AA9) under diversifying selection for successful pathogenesis. Although functional validation is required, it is interesting that at least one gene in a genomic region contributing to population differentiation is related to pathogenesis and might suggest differences in virulence levels between *R. solani* AG1‐IA isolates from Arkansas and Louisiana. Moreover, this is a notable point of convergence between our study and that of Francis et al. (2023), where both highlight the role of genes involved in protein modification and transport, suggesting that these functions might be critical adaptive responses in these agroecosystems. Interestingly, other studies have provided evidence from South American populations suggesting a relatively recent host expansion where the pathogen shifted from rice‐infecting populations to new hosts like *Urochloa* (signalgrass) (Chavarro Mesa et al. 2015). Our finding of high absolute divergence and population‐specific peaks of *F*_ST_ on chromosomes 3 and 8 may reflect similar regional adaptive shifts, though further genomic sampling from other regions is required to conclusively link these local patterns to global invasion routes.

The high genetic diversity of the pathogen in rice and soybean fields may explain, in part, the difficulty of disease control over the years. Moreover, the diseases might present themselves as a disease complex, with sheath spot being difficult to differentiate from sheath blight in rice fields, and aerial and web blight occurring simultaneously in soybean fields. Since *R. solani* and its diseases are predominantly present in rice fields, sheath spot caused by *R. oryzae* is often misclassified and misdiagnosed. Similarly, *R. solani* AG1‐IA is more predominant than *R. solani* AG1‐IB in soybean fields (Rupe and Spurlock 2016). During the screening of several fields in 2021, two *R. oryzae* isolates were obtained from sheath blight/sheath spot rice samples, and no *R. solani* AG1‐IB isolates were recovered from soybean samples. Similary, Rodriguez‐Herrera et al. (2024) did not find AG1‐IB in Louisiana. This highlights the importance of monitoring pathogen populations and their diversity in the fields, and the development of molecular techniques to identify different *Rhizoctonia* species and anastomosis groups.

Overall, there is wide agreement in the literature that *R. solani* AG1‐IA has a mixed reproductive system (e.g., Chavarro Mesa et al. 2015; de Assis et al. 2008). This was strongly supported by our genome‐wide linkage disequilibrium (LD) analysis. We observed a rapid decay in LD, with *r* ^*2*^ values dropping to half‐maximum within 900–1000 bp. This fine‐scale genomic evidence corroborates earlier studies using SSR markers that suggested high levels of recombination in rice‐ and soybean‐infecting populations. The low *F*_IS_ value (average 0.08) that we estimated further confirms that high levels of outcrossing are a consistent feature of *R. solani* AG1‐IA subpopulations, regardless of the host or geographical origin.

In conclusion, this research improves our understanding of the complex dynamics of *R. solani* AG1‐IA, shedding light on its genetic variability, population structure and potential factors driving its genetic differentiation. This knowledge is vital for the development of effective strategies to manage sheath blight in rice and aerial blight in soybeans, as well as related diseases in other crops, ultimately contributing to the sustainability of agricultural practices worldwide. Further research is needed to explore the genetic and environmental factors that influence the pathogen's behaviour and reproductive strategies, potentially leading to more targeted control measures in the future.

## Author Contributions

A.R. and J.G. conceived the study. J.G. performed the research and analysed data. P.G. developed custom scripts for data processing and analysis. P.G. and A.R. contributed to the data analyses. K.R.‐H., V.C., F.D.L, X.‐G.Z., S.T.‐S., T.S. and J.C.C. provided pathogen isolates to build the population. J.G. wrote the manuscript with input from A.R. and P.G. All authors edited and approved the manuscript.

## Funding

This work was supported by Albertine Foundation–Transatlantic Research Partnership and the Arkansas Rice Promotion Board, University of Arkansas faculty startup funding, and supported by the multistate hatch project S1083 at the University of Arkansas ARK02628 and Michigan State University MICL12074.

## Conflicts of Interest

The authors declare no conflicts of interest.

## Supporting information


**Figure S1:** Geographical sample distribution.
**Figure S2:** Distributions of the major allele frequencies of four *Rhizoctonia solani* AG1‐IA isolates. (A) Soybean isolate from Louisiana (AC25). (B) Rice isolate from Arkansas (VC86). (C) Rice isolate from Louisiana (LSU2201R‐04). (D) Soybean isolate from Arkansas (JG69).
**Figure S3:** Ancestry proportions in *K* clusters. Isolates are grouped by location and then host within location.
**Figure S4:** Ancestry proportions in *K* clusters. Isolates are grouped by location and then host and year within location.
**Figure S5:** Identification of clonal isolates in *Rhizoctonia solani* AG1‐IA population. (A). Phylogenetic network before clone correction indicating population subdivision according to location (Arkansas = grey, Texas = Green, Louisiana and Cuba = no colour). Five branches show closely associated isolates within each location. (B). Multilocus genotypes (MLGs) shown by location and host. Only the largest MLG (*n* = 18) had genotypes that crossed subpopulations, namely MLG136 highlighted in yellow. Year of isolate collection was not relevant (data not shown).
**Figure S6:** Test of significance of AMOVA with 999 permutations only for the two largest subpopulations (Arkansas and Louisiana) and the main hosts (rice and soybean).
**Figure S7:** Over‐representation analysis of gene ontologies of 419 genes in genomic regions with *F*_ST_ values > 0.2 (top 5%).
**Figure S8:** LD decay curve of two *R. solani* AG1‐IA subpopulations measured by the squared correlation coefficient (*r* ^2^) between pairs of SNPs plotted against the physical distance of the SNPs in the genome.
**Figure S9:** Overall alignment rates of mapped reads of a subsample of 48 isolates to three different reference genomes of Rhizoctonia solani AG1‐IA. Strains XN and HG81 from China and B2 from the United States.
**Table S1:** List of *Rhizoctonia solani* AG1‐IA isolates.
**Table S2:**. Analysis of molecular variance in *Rhizoctonia solani* AG1‐IA population.

## Data Availability

Genetic data: Raw sequence reads are deposited in the SRA under BioProject ID PRJNA1292868. Sample metadata: Metadata are also stored in the SRA (BioProject PRJNA1292868). Code: Scripts are available in GitHub project: https://github.com/JuanitaGil/Rhizoctonia‐solani‐AG1‐IA‐population‐genomics/tree/main.
