# Supplementary material for: Population Genomic Analyses Reveal Geographic Structure in Rhizoctonia solani AG1‐IA Isolates Associated With Agronomic Crops
Source: Mol Ecol. 2026 Apr 27;35:e70359. doi: 10.1111/mec.70359 (PMC13113206; doi:10.1111/mec.70359)
Supplement: Supplementary file 1 — Figure S1: Geographical sample distribution. Figure S2: Distributions of the major allele frequencies of four Rhizoctonia solani AG1‐IA isolates. (A) Soybean isolate from Louisiana (AC25). (B) Rice isolate from Arkansas (VC86). (C) Rice isolate from Louisiana (LSU2201R‐04). (D) Soybean isolate from Arkansas (JG69). Figure S3: Ancestry proportions in K clusters. Isolates are grouped by location and then host within location. Figure S4: Ancestry proportions in K clusters. Isolates are grouped by location and then host and year within location. Figure S5: Identification of clonal isolates in Rhizoctonia solani AG1‐IA population. (A). Phylogenetic network before clone correction indicating population subdivision according to location (Arkansas = grey, Texas = Green, Louisiana and Cuba = no colour). Five branches show closely associated isolates within each location. (B). Multilocus genotypes (MLGs) shown by location and host. Only the largest MLG (n = 18) had genotypes that crossed subpopulations, namely MLG136 highlighted in yellow. Year of isolate collection was not relevant (data not shown). Figure S6: Test of significance of AMOVA with 999 permutations only for the two largest subpopulations (Arkansas and Louisiana) and the main hosts (rice and soybean). Figure S7: Over‐representation analysis of gene ontologies of 419 genes in genomic regions with F ST values > 0.2 (top 5%). Figure S8: LD decay curve of two R. solani AG1‐IA subpopulations measured by the squared correlation coefficient (r 2) between pairs of SNPs plotted against the physical distance of the SNPs in the genome. Figure S9: Overall alignment rates of mapped reads of a subsample of 48 isolates to three different reference genomes of Rhizoctonia solani AG1‐IA. Strains XN and HG81 from China and B2 from the United States. Table S1: List of Rhizoctonia solani AG1‐IA isolates. Table S2:. Analysis of molecular variance in Rhizoctonia solani AG1‐IA population. [file MEC-35-e70359-s001.docx]

### **Supplementary tables and figures**

**Supplementary Table S1.** List of *Rhizoctonia solani* AG1-IA isolates.

|  | | Origin | | | | |  |
| --- | --- | --- | --- | --- | --- | --- | --- |
| Isolate ID | **AG^a^** | **Host** | **Location** | **State** | **Country** | **Year** | **Source^b^** |
| Cuba2 | AG1-IA | Common bean | Cuba | - | Cuba | 1993 | A |
| RR0105 | AG1-IA | Rice | Clay | Arkansas | USA | 2001 | A |
| RR0108 | AG1-IA | Rice | Lawrence | Arkansas | USA | 2001 | A |
| RR0125 | AG1-IA | Rice | Arkansas | Arkansas | USA | 2001 | A |
| RR0134 | AG1-IA | Rice | Faulkner | Arkansas | USA | 2001 | A |
| VC104 | AG1-IA | Rice | - | Arkansas | USA | 2004 | A |
| VC12 | AG1-IA | Bermuda grass | - | - | - | 2002 | A |
| VC123 | AG1-IA | Rice | - | Arkansas | USA | 2004 | A |
| VC14 | AG1-IA | Rice | Randolph | Arkansas | USA | 2005 | A |
| VC142 | AG1-IA | Rice | Randolph | Arkansas | USA | 2005 | A |
| VC163 | AG1-IA | Rice | Acadia Parish | Louisiana | USA | 2008 | A |
| VC164 | AG1-IA | Rice | Acadia Parish | Louisiana | USA | 2008 | A |
| VC166 | AG1-IA | Rice | Acadia Parish | Louisiana | USA | 2008 | A |
| VC170 | AG1-IA | Rice | Acadia Parish | Louisiana | USA | 2008 | A |
| VC180 | AG1-IA | Rice | Acadia Parish | Louisiana | USA | 2008 | A |
| VC189 | AG1-IA | Rice | Lonoke | Arkansas | USA | 2008 | A |
| VC210 | AG1-IA | Rice | Acadia Parish | Louisiana | USA | 2008 | A |
| VC211 | AG1-IA | Rice | Crowley | Louisiana | USA | 2008 | A |
| VC215 | AG1-IA | Rice | Ashley | Arkansas | USA | 2008 | A |
| VC217 | AG1-IA | Rice | Ashley | Arkansas | USA | 2008 | A |
| VC220 | AG1-IA | Rice | Ashley | Arkansas | USA | 2008 | A |
| VC23 | AG1-IA | Corn | Clark | Arkansas | USA | 2005 | A |
| VC230 | AG1-IA | Rice | Lincoln | Arkansas | USA | 2008 | A |
| VC248 | AG1-IA | Rice | Lincoln | Arkansas | USA | 2008 | A |
| VC288 | AG1-IA | Rice | Jefferson | Arkansas | USA | 2008 | A |
| VC299 | AG1-IA | Rice | Jefferson | Arkansas | USA | 2008 | A |
| VC319 | AG1-IA | Rice | Arkansas | Arkansas | USA | 2008 | A |
| VC33 | AG1-IA | Rice | Independence | Arkansas | USA | 1999 | A |
| VC339 | AG1-IA | Rice | Arkansas | Arkansas | USA | 2008 | A |
| VC349 | AG1-IA | Rice | Prairie | Arkansas | USA | 2008 | A |
| VC379 | AG1-IA | Rice | Lonoke | Arkansas | USA | 2008 | A |
| VC387 | AG1-IA | Rice | Arkansas | Arkansas | USA | 2008 | A |
| VC449 | AG1-IA | Rice | Jefferson | Texas | USA | 2008 | A |
| VC458 | AG1-IA | Rice | Jefferson | Texas | USA | 2008 | A |
| VC459 | AG1-IA | Rice | Acadia Parish | Louisiana | USA | 2008 | A |
| VC470 | AG1-IA | Sorghum | Arkansas | Arkansas | USA | 2009 | A |
| VC50 | AG1-IA | Soybean | Clark | Arkansas | USA | 2000 | A |
| VC70 | AG1-IA | Rice | Crittenden | Arkansas | USA | 2005 | A |
| VC74 | AG1-IA | Rice | Crittenden | Arkansas | USA | 2005 | A |
| VC80 | AG1-IA | Rice | - | Arkansas | USA | 2005 | A |
| VC86 | AG1-IA | Rice | Crittenden | Arkansas | USA | 2005 | A |
| 117T | AG1-IA | Soybean | Arkansas | Arkansas | USA | 2020 | B |
| JX | AG1-IA | Soybean | Arkansas | Arkansas | USA | 2020 | B |
| Q1 | AG1-IA | Soybean | Arkansas | Arkansas | USA | 2020 | B |
| Q17 | AG1-IA | Soybean | Arkansas | Arkansas | USA | 2020 | B |
| Q19 | AG1-IA | Soybean | Arkansas | Arkansas | USA | 2020 | B |
| Q21 | AG1-IA | Soybean | Arkansas | Arkansas | USA | 2020 | B |
| Q22 | AG1-IA | Soybean | Arkansas | Arkansas | USA | 2020 | B |
| Q24 | AG1-IA | Soybean | Arkansas | Arkansas | USA | 2020 | B |
| Q25 | AG1-IA | Soybean | Arkansas | Arkansas | USA | 2020 | B |
| Q26 | AG1-IA | Soybean | Arkansas | Arkansas | USA | 2020 | B |
| Q29 | AG1-IA | Soybean | Arkansas | Arkansas | USA | 2020 | B |
| Q30 | AG1-IA | Soybean | Arkansas | Arkansas | USA | 2020 | B |
| Q4 | AG1-IA | Soybean | Arkansas | Arkansas | USA | 2020 | B |
| Q5 | AG1-IA | Soybean | Arkansas | Arkansas | USA | 2020 | B |
| QBS26 | AG1-IA | Soybean | Arkansas | Arkansas | USA | 2020 | B |
| AC2 | AG1-IA | Soybean | Acadia Parish | Louisiana | USA | 2021 | C |
| AG1-IA | AG1-IA | Soybean | - | - | - | - | C |
| JG30 | AG1-IA | Rice | Monroe | Arkansas | USA | 2021 | C |
| JG56 | AG1-IA | Rice | PTRS | Arkansas | USA | 2021 | C |
| JG57 | AG1-IA | Rice | PTRS | Arkansas | USA | 2021 | C |
| JG60 | AG1-IA | Rice | PTRS | Arkansas | USA | 2021 | C |
| JG62 | AG1-IA | Rice | PTRS | Arkansas | USA | 2021 | C |
| JG65 | AG1-IA | Rice | PTRS | Arkansas | USA | 2021 | C |
| JG69 | AG1-IA | Soybean | Arkansas | Arkansas | USA | 2021 | C |
| JG7 | AG1-IA | Rice | Arkansas | Arkansas | USA | 2021 | C |
| Rs2 | AG1-IA | Soybean | St. Francis | Arkansas | USA | 2022 | C |
| Rs3 | AG1-IA | Soybean | St. Francis | Arkansas | USA | 2022 | C |
| Rs4 | AG1-IA | Soybean | St. Francis | Arkansas | USA | 2022 | C |
| Rs5 | AG1-IA | Soybean | St. Francis | Arkansas | USA | 2022 | C |
| Rs6 | AG1-IA | Soybean | Drew | Arkansas | USA | 2022 | C |
| Rs7 | AG1-IA | Soybean | Drew | Arkansas | USA | 2022 | C |
| Rs8 | AG1-IA | Soybean | Ashley | Arkansas | USA | 2022 | C |
| AC13 | AG1-IA | Soybean | Acadia Parish | Louisiana | USA | 2021 | D |
| AC14 | AG1-IA | Soybean | Acadia Parish | Louisiana | USA | 2021 | D |
| AC15 | AG1-IA | Soybean | Acadia Parish | Louisiana | USA | 2021 | D |
| AC16 | AG1-IA | Soybean | Acadia Parish | Louisiana | USA | 2021 | D |
| AC18 | AG1-IA | Soybean | Acadia Parish | Louisiana | USA | 2021 | D |
| AC19 | AG1-IA | Soybean | Acadia Parish | Louisiana | USA | 2021 | D |
| AC20 | AG1-IA | Soybean | Acadia Parish | Louisiana | USA | 2021 | D |
| AC21 | AG1-IA | Soybean | Acadia Parish | Louisiana | USA | 2021 | D |
| AC22 | AG1-IA | Soybean | Acadia Parish | Louisiana | USA | 2021 | D |
| AC23 | AG1-IA | Soybean | Acadia Parish | Louisiana | USA | 2021 | D |
| AC24 | AG1-IA | Soybean | Acadia Parish | Louisiana | USA | 2021 | D |
| AC25 | AG1-IA | Soybean | Acadia Parish | Louisiana | USA | 2021 | D |
| AC28 | AG1-IA | Soybean | Acadia Parish | Louisiana | USA | 2021 | D |
| AC29 | AG1-IA | Soybean | Acadia Parish | Louisiana | USA | 2021 | D |
| AC5 | AG1-IA | Soybean | Acadia Parish | Louisiana | USA | 2021 | D |
| AC6 | AG1-IA | Soybean | Acadia Parish | Louisiana | USA | 2021 | D |
| AC8 | AG1-IA | Soybean | Acadia Parish | Louisiana | USA | 2021 | D |
| AC9 | AG1-IA | Soybean | Acadia Parish | Louisiana | USA | 2021 | D |
| AV1 | AG1-IA | Soybean | Avoyelles Parish | Louisiana | USA | 2021 | D |
| AV11 | AG1-IA | Soybean | Avoyelles Parish | Louisiana | USA | 2021 | D |
| AV13 | AG1-IA | Soybean | Avoyelles Parish | Louisiana | USA | 2021 | D |
| AV14 | AG1-IA | Soybean | Avoyelles Parish | Louisiana | USA | 2021 | D |
| AV15 | AG1-IA | Soybean | Avoyelles Parish | Louisiana | USA | 2021 | D |
| AV17 | AG1-IA | Soybean | Avoyelles Parish | Louisiana | USA | 2021 | D |
| AV19 | AG1-IA | Soybean | Avoyelles Parish | Louisiana | USA | 2021 | D |
| AV21 | AG1-IA | Soybean | Avoyelles Parish | Louisiana | USA | 2021 | D |
| AV3 | AG1-IA | Soybean | Avoyelles Parish | Louisiana | USA | 2021 | D |
| AV6 | AG1-IA | Soybean | Avoyelles Parish | Louisiana | USA | 2021 | D |
| AV9 | AG1-IA | Soybean | Avoyelles Parish | Louisiana | USA | 2021 | D |
| Avoy1 | AG1-IA | Soybean | Avoyelles Parish | Louisiana | USA | 2020 | D |
| Avoy2 | AG1-IA | Soybean | Avoyelles Parish | Louisiana | USA | 2020 | D |
| Avoy5 | AG1-IA | Soybean | East Baton Rouge | Louisiana | USA | 2020 | D |
| ML1 | AG1-IA | Soybean | Acadia Parish | Louisiana | USA | 2020 | D |
| RT1 | AG1-IA | Soybean | Acadia Parish | Louisiana | USA | 2020 | D |
| WS4 | AG1-IA | Soybean | Acadia Parish | Louisiana | USA | 2020 | D |
| TX-MS1 | AG1-IA | Soybean | Chambers | Texas | USA | 2013 | E |
| TX-RS1 | AG1-IA | Rice | Jefferson | Texas | USA | 2010 | E |
| TX-RS1-1 | AG1-IA | Rice | Jefferson | Texas | USA | 2010 | E |
| TX-RS1-2 | AG1-IA | Rice | Jefferson | Texas | USA | 2010 | E |
| TX-RS12 | AG1-IA | Rice | Jefferson | Texas | USA | 2018 | E |
| TX-RS13 | AG1-IA | Rice | Wharton | Texas | USA | 2018 | E |
| TX-RS2 | AG1-IA | Rice | Jefferson | Texas | USA | 2010 | E |
| TX21-1 | AG1-IA | Rice | Colorado | Texas | USA | 2021 | E |
| TX21-3 | AG1-IA | Rice | Colorado | Texas | USA | 2021 | E |
| TX21-5 | AG1-IA | Rice | Colorado | Texas | USA | 2021 | E |
| TX21-6 | AG1-IA | Rice | Colorado | Texas | USA | 2021 | E |
| TX21-7 | AG1-IA | Rice | Liberty | Texas | USA | 2021 | E |
| TX21-8 | AG1-IA | Rice | Colorado | Texas | USA | 2021 | E |
| TX21-9 | AG1-IA | Rice | Colorado | Texas | USA | 2021 | E |
| LR-12-2 | AG1-IA | Rice | Acadia Parish | Louisiana | USA | 2012 | F |
| LR-12-3 | AG1-IA | Rice | Acadia Parish | Louisiana | USA | 2012 | F |
| LSU2201R-01 | AG1-IA | Rice | Acadia Parish | Louisiana | USA | 2022 | F |
| LSU2201R-02 | AG1-IA | Rice | Acadia Parish | Louisiana | USA | 2022 | F |
| LSU2201R-03 | AG1-IA | Rice | Acadia Parish | Louisiana | USA | 2022 | F |
| LSU2201R-04 | AG1-IA | Rice | Acadia Parish | Louisiana | USA | 2022 | F |
| LSU2201R-05 | AG1-IA | Rice | Acadia Parish | Louisiana | USA | 2022 | F |
| LSU2201R-06 | AG1-IA | Rice | Acadia Parish | Louisiana | USA | 2022 | F |
| LSU2201R-07 | AG1-IA | Rice | Acadia Parish | Louisiana | USA | 2022 | F |
| LSU2201S-08 | AG1-IA | Soybean | Acadia Parish | Louisiana | USA | 2022 | F |
| LSU2203R-01 | AG1-IA | Rice | Vermilion Parish | Louisiana | USA | 2022 | F |
| LSU2204R-02 | AG1-IA | Rice | Evangeline Parish | Louisiana | USA | 2022 | F |
| LSU2204R-03 | AG1-IA | Rice | Evangeline Parish | Louisiana | USA | 2022 | F |
| LSU2204R-04 | AG1-IA | Rice | Evangeline Parish | Louisiana | USA | 2022 | F |
| LSU2204R-05 | AG1-IA | Rice | Evangeline Parish | Louisiana | USA | 2022 | F |
| LSU2204R-06 | AG1-IA | Rice | Evangeline Parish | Louisiana | USA | 2022 | F |
| LSU2205R-02 | AG1-IA | Rice | St Landry Parish | Louisiana | USA | 2022 | F |
| LSU2205R-03 | AG1-IA | Rice | St Landry Parish | Louisiana | USA | 2022 | F |
| LSU2205R-04 | AG1-IA | Rice | St Landry Parish | Louisiana | USA | 2022 | F |
| LSU2207R-01 | AG1-IA | Rice | Avoyelles Parish | Louisiana | USA | 2022 | F |
| LSU2207R-02 | AG1-IA | Rice | Avoyelles Parish | Louisiana | USA | 2022 | F |
| LSU2207S-03 | AG1-IA | Soybean | Avoyelles Parish | Louisiana | USA | 2022 | F |
| LSU2211R-01 | AG1-IA | Rice | Acadia Parish | Louisiana | USA | 2022 | F |

^a^Anastomosis Group

^b^Isolate source: A=Dr. Correll ENPL U of A; B=Dr. Spurlock U of A; C=This study; D=Dr. Thomas-Sharma LSU; E=Dr. Zhou Texas A&M AgriLife Research Center; F=Dr. Dalla Lana LSU

**Supplementary Table S2.** Analysis of molecular variance in *Rhizoctonia solani* AG1-IA population.

| **Test** | **Sigma (Obs)** | **Std. Obs** | **%** | **Phi** | **P value** |
| --- | --- | --- | --- | --- | --- |
| Variations within samples | 165463.1013 | -2.472 | 89.726 | 0.113 | 0.009 |
| Variations between samples | -2185.3246 | -0.667 | -1.185 | -0.002 | 0.765 |
| Variations between host | 392.8785 | 3.422 | 0.213 | -0.013 | 0.002 |
| Variations between location | 20738.8012 | 1.343 | 11.246 | 0.103 | 0.357 |


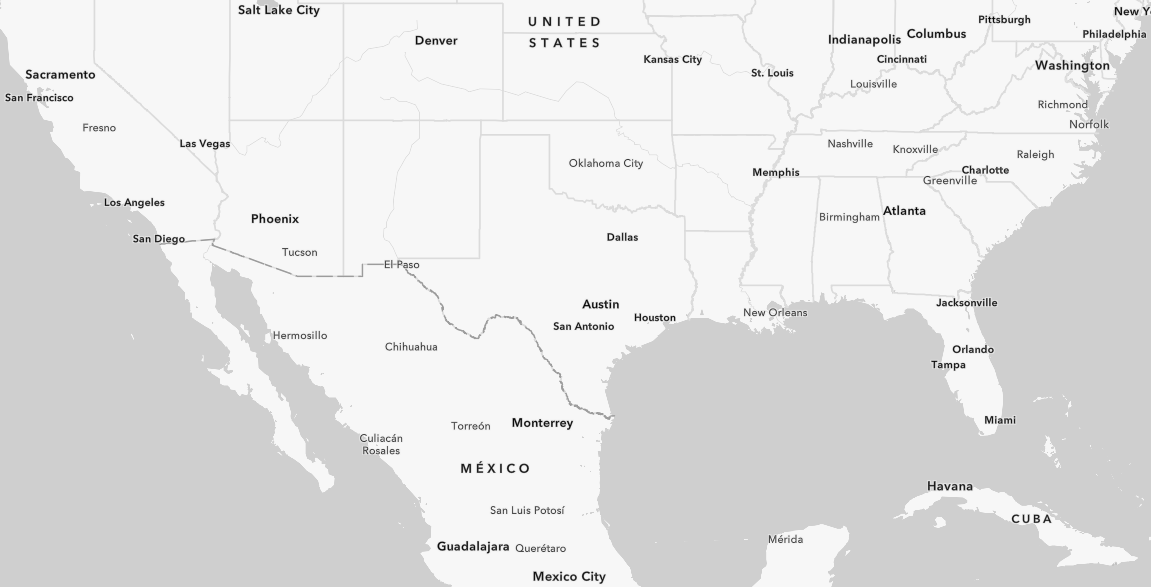

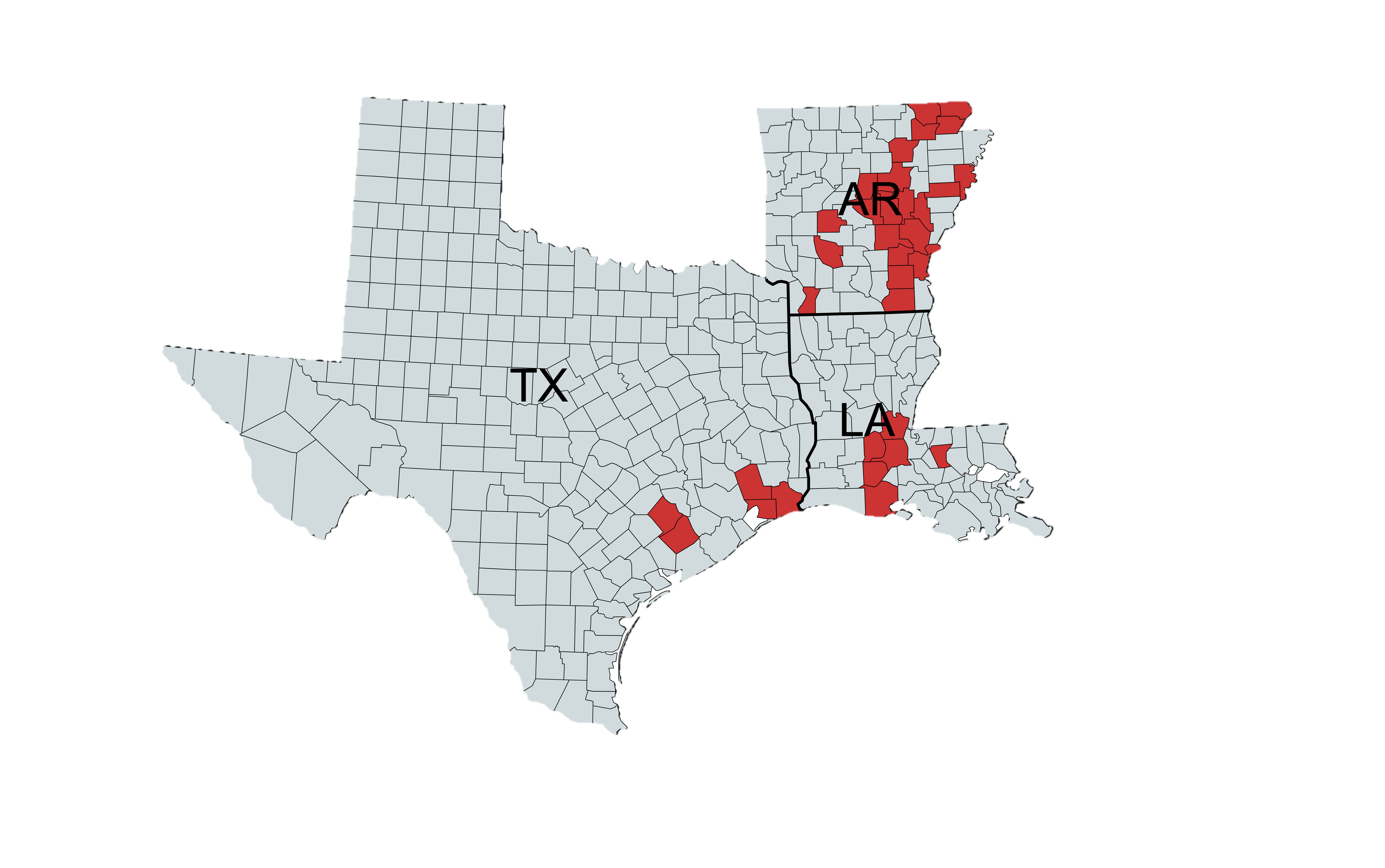

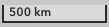

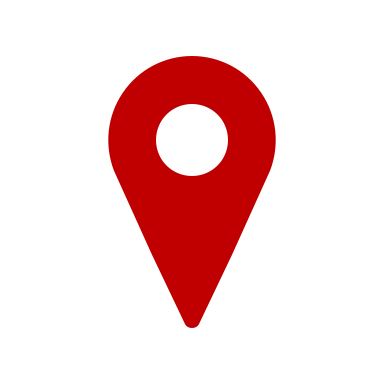


**Supplementary Figure S1.** Geographical sample distribution.


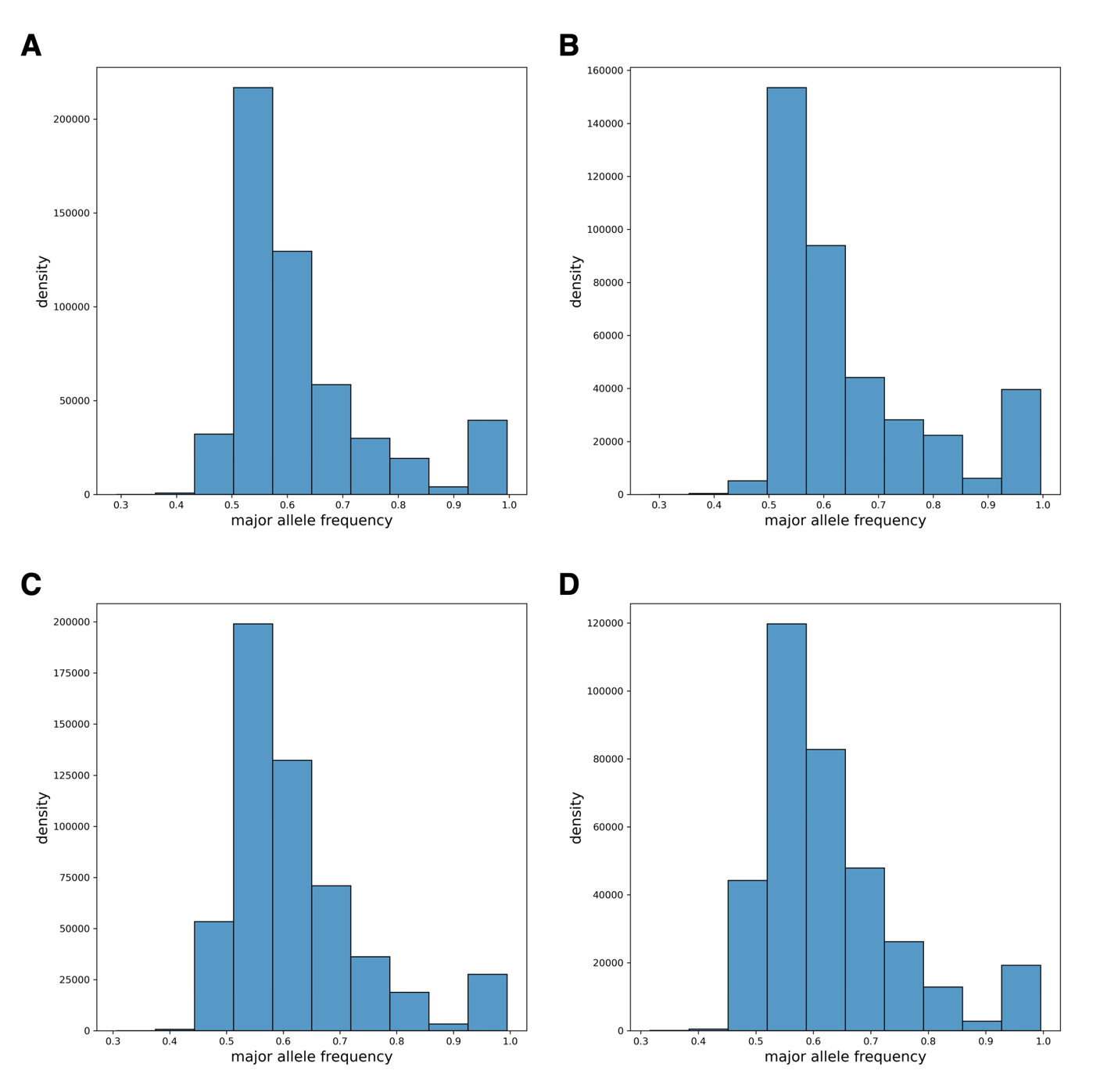


**Supplementary Figure S2.** Distributions of the major allele frequencies of four *Rhizoctonia solani* AG1-IA isolates. **A**. Soybean isolate from Louisiana (AC25). **B**. Rice isolate from Arkansas (VC86). **C**. Rice isolate from Louisiana (LSU2201R-04). **D**. Soybean isolate from Arkansas (JG69).


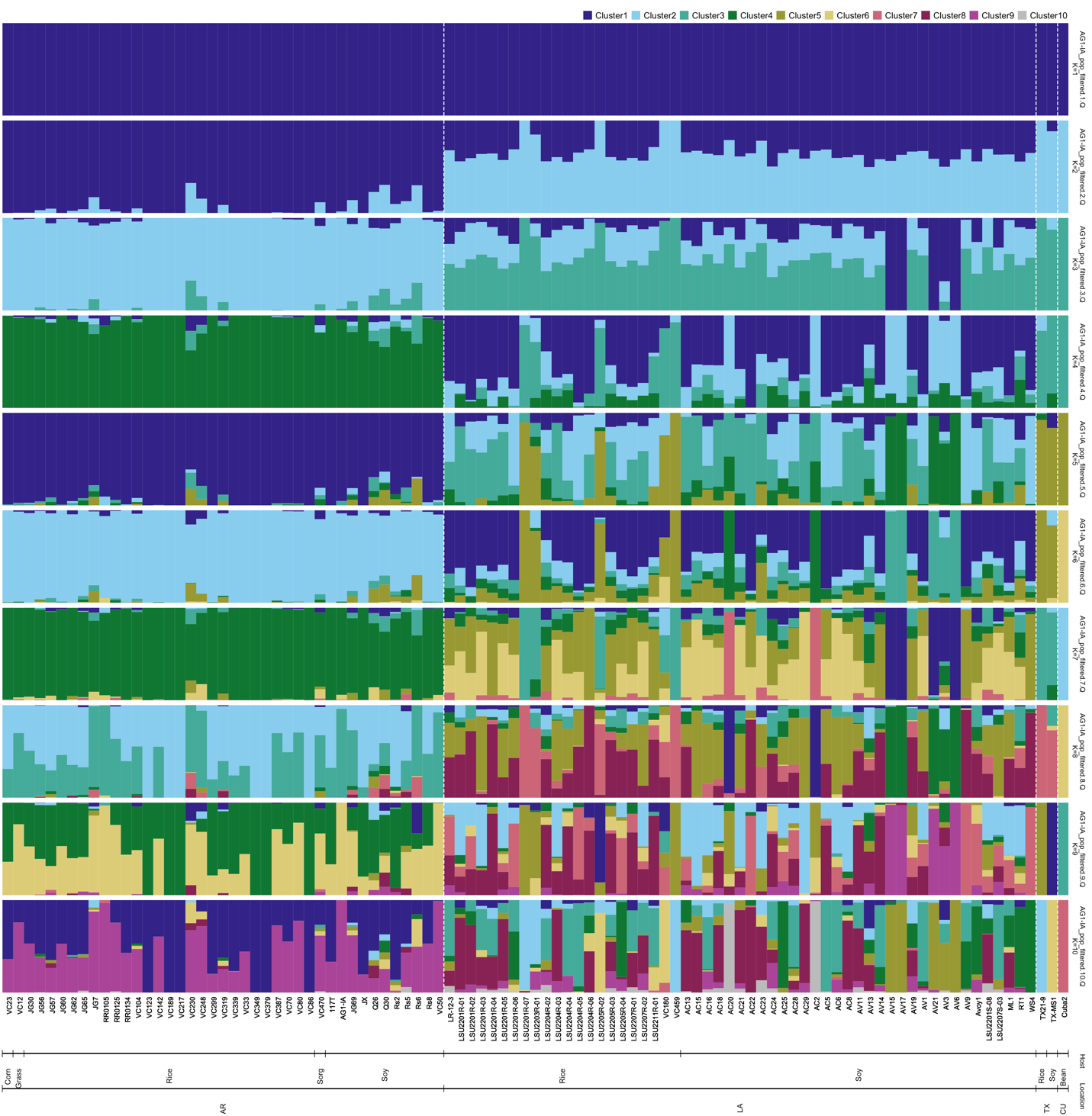


**Supplementary Figure S3**. Ancestry proportions in K clusters. Isolates are grouped by location and then host within location.


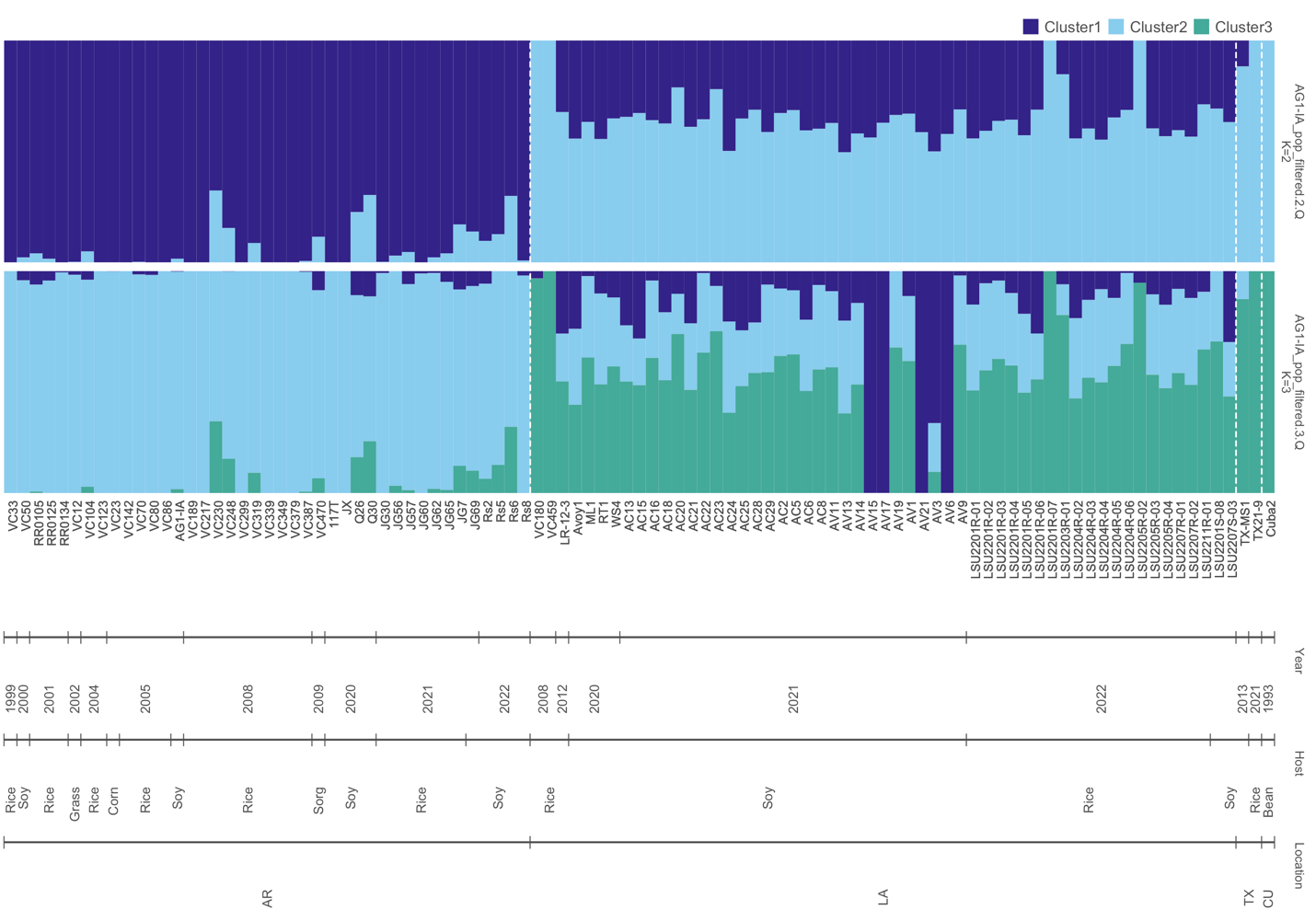


**Supplementary Figure S4**. Ancestry proportions in K clusters. Isolates are grouped by location and then host and year within location.

**B**

**A**


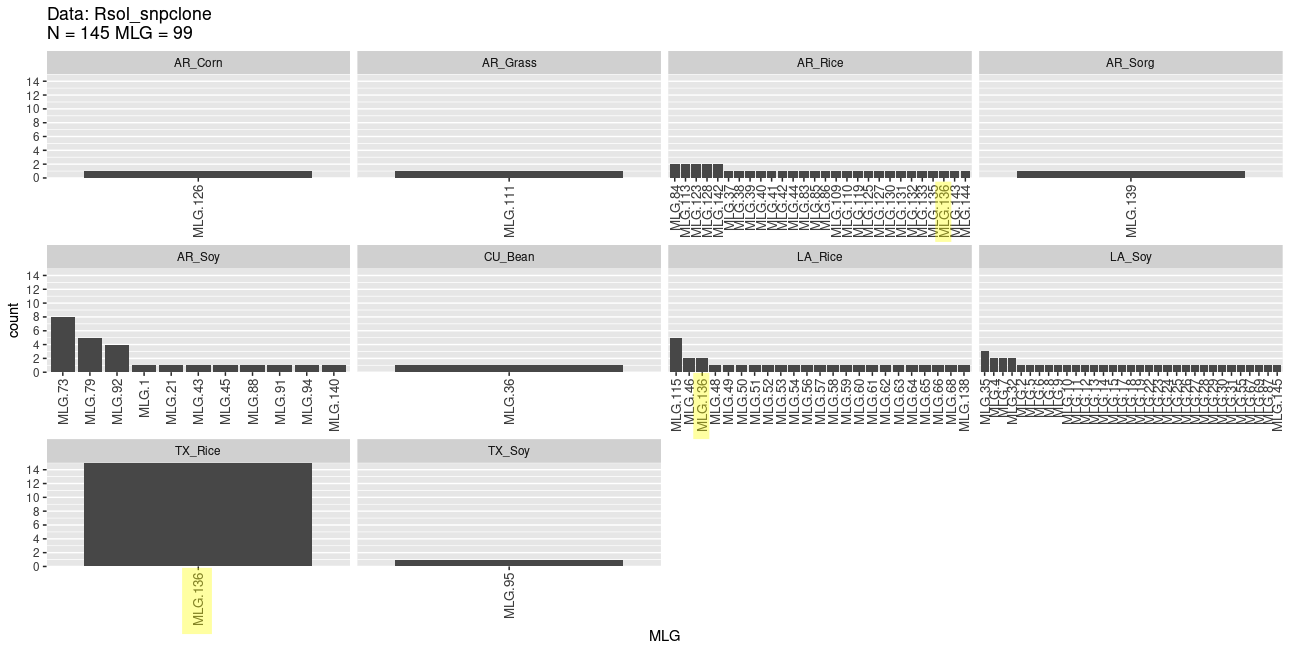


**TX**

**AR**

**LA**

**LA**

**Supplementary Figure S5.** Identification of clonal isolates in *Rhizoctonia solani* AG1-IA population. **A)**. Phylogenetic network before clone correction indicating population subdivision according to location (Arkansas = grey, Texas = Green, Louisiana and Cuba = no color). Five branches show closely associated isolates within each location. **B)**. Multilocus genotypes (MLGs) shown by location and host. Only the largest MLG (n=18) had genotypes that crossed subpopulations, namely MLG136 highlighted in yellow. Year of isolate collection was not relevant (data not shown).


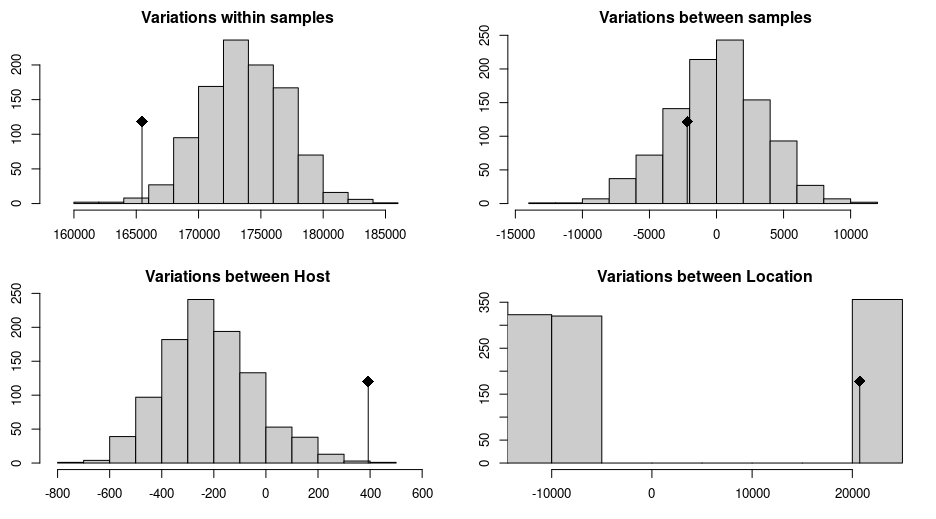


**Supplementary Figure S6.** Test of significance of AMOVA with 999 permutations only for the two largest subpopulations (Arkansas and Louisiana) and the main hosts (rice and soybean).


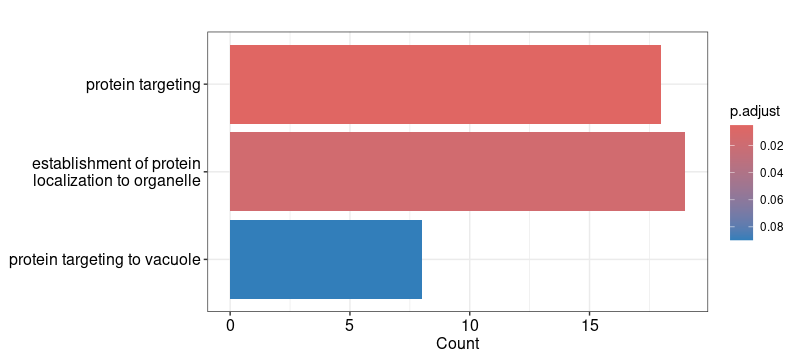


**Supplementary Figure S7.** Over-representation analysis of gene ontologies of 419 genes in genomic regions with F_ST_ values > 0.2 (top 5%).

**Supplementary Figure S8.** LD decay curve of two *R. solani* AG1-IA subpopulations measured by the squared correlation coefficient (r^2^) between pairs of SNPs plotted against the physical distance of the SNPs in the genome.


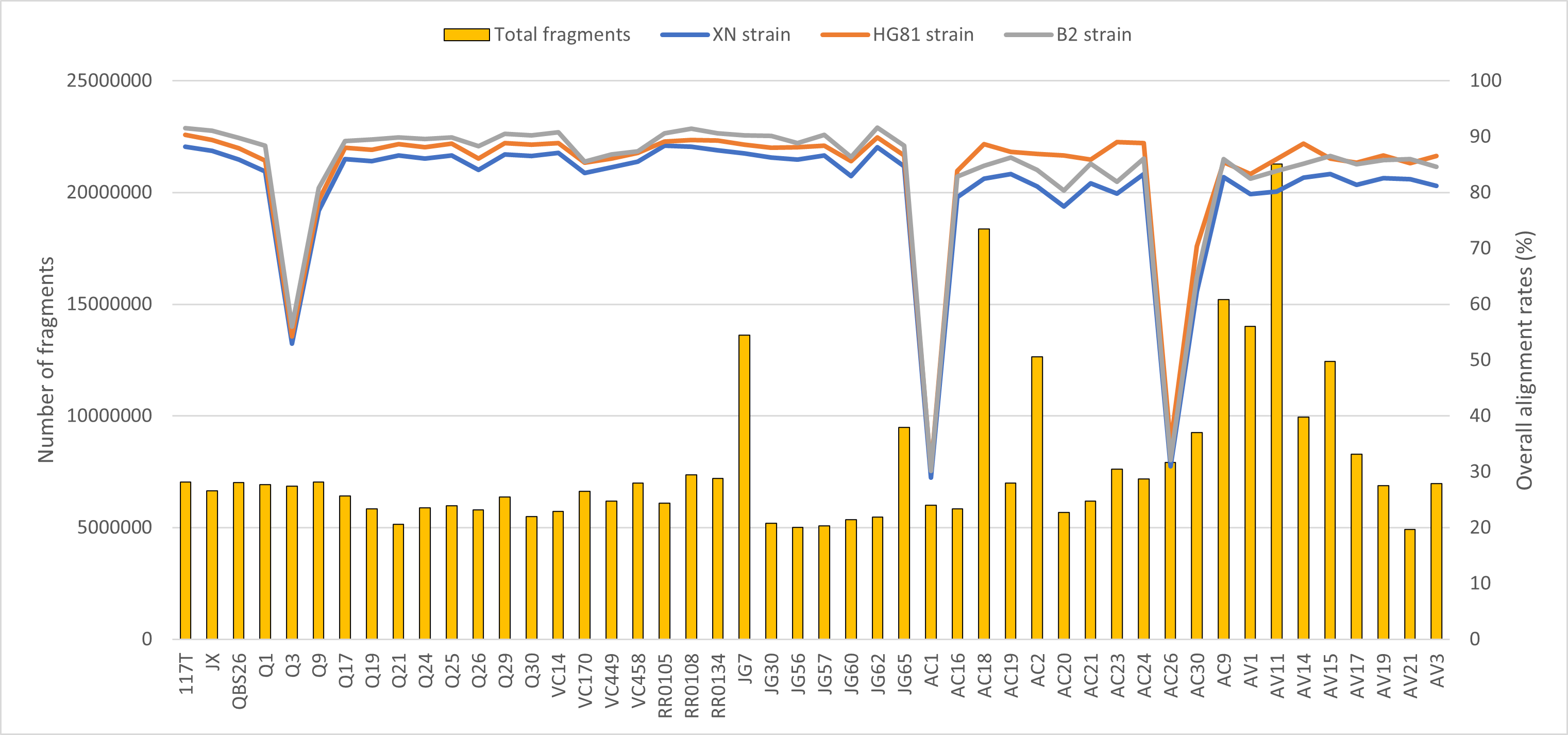
**Supplementary Figure S9**. Overall alignment rates of mapped reads of a subsample of 48 isolates to three different reference genomes of *Rhizoctonia solani* AG1-IA. Strains XN and HG81 from China and B2 from the U.S.
